# HSP47 in human diseases: Navigating pathophysiology, diagnosis and therapy

**DOI:** 10.1002/ctm2.1755

**Published:** 2024-08-12

**Authors:** Essak. S. Khan, Tobias Däinghaus

**Affiliations:** ^1^ Posttranscriptional Gene Regulation Cancer Research and Experimental Hemostasis University Medical Center Mainz (UMCM) Mainz Germany; ^2^ Center for Thrombosis and Hemostasis (CTH) UMCM Mainz Germany; ^3^ German Consortium for Translational Cancer Research (DKTK) DKFZ Frankfurt‐Mainz Frankfurt am Main Germany

**Keywords:** biomarker, heat shock protein 47 (HSP47), human disorders, therapy

## Abstract

Heat shock protein 47 (HSP47) is a chaperone protein responsible for regulating collagen maturation and transport, directly impacting collagen synthesis levels. Aberrant HSP47 expression or malfunction has been associated with collagen‐related disorders, most notably fibrosis. Recent reports have uncovered new functions of HSP47 in various cellular processes. Hsp47 dysregulation in these alternative roles has been linked to various diseases, such as cancer, autoimmune and neurodegenerative disorders, thereby highlighting its potential as both a diagnostic biomarker and a therapeutic target. In this review, we discuss the pathophysiological roles of HSP47 in human diseases, its potential as a diagnostic tool, clinical screening techniques and its role as a target for therapeutic interventions.

## Graphical abstract

• HSP47 plays a crucial role in collagen biosynthesis and tissue‐specific functions, contributing to fibrosis and collagen‐related disorders.

• Beyond collagen assembly, it is involved in diverse cellular processes like ER stress, angiogenesis, metastasis and thrombosis.

• HSP47 dysfunction is associated with various cancers, diabetes and neurodegenerative diseases.

• HSP47 serves as a versatile biomarker and therapeutic target across a wide range of pathologies.

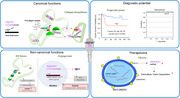

## 1 INTRODUCTION

Molecular chaperones are essential for maintaining cellular proteostasis by intricately guiding proteins folding and refolding processes, delicately balancing the functionality of over 20 000 proteins in humans.^1^ The aberrant function of these molecular chaperones is often characterized as either loss‐of‐function or toxic gain‐of‐function contributing to human diseases.^2^ Heat shock protein 47 (HSP47), a molecular chaperone, unfolds a narrative far beyond its initial collagen‐centric role.^3^ It is traditionally understood as a collagen‐specific molecular player involved in collagen folding, quality control and assembly inside the cells.^4^, ^5^ It has a constitutive expression with synthesized collagen, which helps in tissue‐specific functions.^5^, ^6^ Aberrant expression or function of HSP47 is involved in collagenopathies.^4^, ^5^, ^6^ It can either be linked to mutation in HSP47 itself leading to defective collagen production in diseases like osteogenesis imperfecta (OI) and epidermolysis bullosa or due to overexpression of collagen in fibrosis.^3^ Beyond its canonical role, it interacts with other cellular processes, and new functions have been discovered in promoting various disease like cancers,^7^, ^8^, ^9^ autoimmune diseases (diabetes type 1^10^) and neurodegenerative diseases.^11^ Furthermore, recent reports highlight its diagnostic potential as a biomarker^12^ and therapeutic target.^13^ Here we shed light on the pathophysiological role of HSP47, discuss its diagnostic potential and techniques used to screen it for clinical implementation and its role in therapy.^1^, ^2^, ^3^, ^4^, ^5^, ^6^, ^7^, ^8^, ^9^, ^10^, ^11^, ^12^, ^13^

## 2 ROLES OF HSP47 IN PATHOPHYSIOLOGY

### 2.1 Canonical roles of HSP47

#### 2.1.1 Collagen as a classical client

**FIGURE 1:**
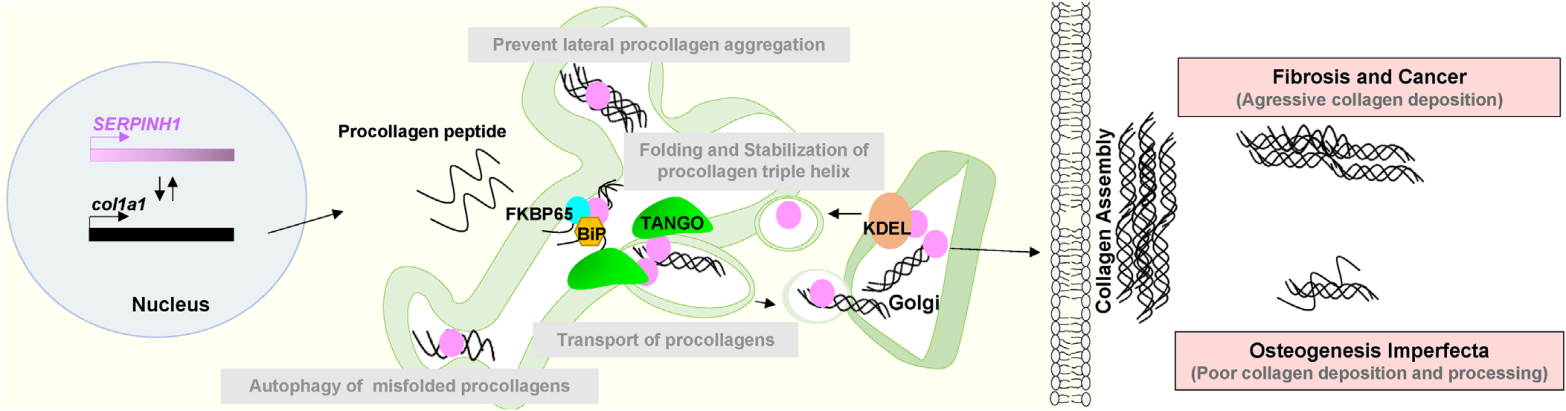
Heat shock protein 47 (HSP47) in collagen biosynthesis. HSP47 (Pink), a chaperone protein expressed by *SERPINH1*, facilitates collagen (COL1a1) folding and stabilization in the endoplasmic reticulum (ER).^3^, ^4^ Interacting with chaperones (FKBP65 (Blue), binding immunoglobulin protein (BiP) (Yellow)), it regulates collagen posttranslational modifications, prevents misfolded collagen aggregation and aids in procollagen transport to the Golgi via Transport and Golgi Organization (TANGO1) interaction.^23^, ^26^ Elevated HSP47 levels promote aggressive collagen deposition in fibrosis^48^, ^49^ and cancer,^59^ whereas low levels cause poor collagen processing in osteogenesis imperfecta.^83^

HSP47 functions as a collagen‐specific molecular chaperone.^3^, ^4^ It is involved in various essential functions related to collagen biosynthesis and maturation, comprehensively explained elsewhere (Figure 1).^3^, ^14^, ^15^ Once procollagen peptides enter the endoplasmic reticulum (ER) and the initial triple helices form, HSP47 binds to the triple‐helical structure, stabilizing and further folding the protein.^16^, ^17^ Alongside other chaperones, it prevents procollagen aggregation, thereby promoting quality control of misfolded protein.^16^, ^18^, ^19^, ^20^ HSP47 facilitates the transport of folded procollagen from the ER to the Golgi apparatus, dissociates procollagens and is then recruited back to the ER via KDEL receptors.^5^, ^21^ Constitutive expression of HSP47 with collagen synthesis has been shown to contribute to tissue‐specific functions.^3^, ^6^, ^10^, ^13^ Although the mechanism of how HSP47 regulates collagen expression or vice versa is poorly understood, it acts as a critical player in folding and regulating collagen homeostasis.

#### 2.1.2 Interaction with co‐client of collagen during ER exit

HSP47 plays a crucial role in the dynamic interplay with key proteins that helps in the collagen folding and transport machinery. During procollagen folding, it engages in a molecular duet with proteins like FKBP265 (a member of the FK506‐binding protein family) and Transport and Golgi Organization (TANGO).^22^ Recent reports suggest that FKBP65 and HSP47 cooperate in collagen maturation during normal bone development, but this interaction is impaired in mutant HSP47 OI cells.^23^ Additionally, HSP47, FKBP65 and binding immunoglobulin protein (Bip) modulate the activity of lysyl hydroxylase 2 (catalyse hydroxylation of lysines on collagen), contributing to the regulation of connective tissue quality through collagen modification.^24^TANGO1 is involved in loading large extracellular matrix (ECM) proteins like collagens into COPII vesicles for intracellular transport.^25^ It indirectly interacts with collagens through its Src homology 3 domain binding to HSP47, guiding procollagens to COPII vesicles.^26^ In summary, HSP47's role in collagen packaging transcends mere escort duty, positioning it as a central player in collagen biosynthesis.

### 2.2 Non‐canonical roles of HSP47

Recently, new interactions of HSP47 have been discovered beyond its role in collagen assembly in unfolded protein response (UPR), angiogenesis and platelets‐induced thrombosis and haemostasis. These novel interactions may have implications for various cellular processes.

#### 2.2.1 ER stress

**FIGURE 2:**
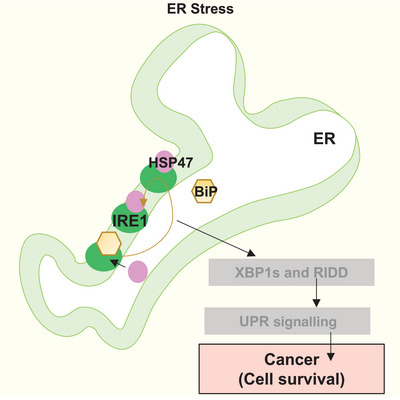
HSP47 regulates unfolded protein response(UPR) in ER stress. It binds toInositol‐requiring enzyme 1 (IRE1), kicking off BiP, and helps IRE1 cluster and activate, which aids in managing ER stress. In cancer, it interacts with calreticulin and IRE1α to help the cells survive and resist chemotherapy by escaping ER stress.^30^

The UPR is a dynamic signalling network that helps maintain the proper functioning of the ER.^27^ Inositol‐requiring enzyme 1 (IRE1), a crucial part of the UPR acts as a sensor to manage the protein‐folding balance in the ER.^28^ Under normal conditions, a chaperone protein called BiP keeps IRE1 in an inactive state. However, when there is stress in the ER due to misfolded proteins, BiP releases IRE1, allowing it to become active. Studies have shown that HSP47 has a strong affinity for IRE1 and displaces BiP in a tug‐of‐war, allowing IRE1 to cluster and become active.^7^, ^29^ In breast and pancreatic cancer, Hsp47 promotes chemoresistance by interacting with calreticulin and IRE1α, thus escaping ER stress^30^ (Figure 2). In simpler terms, HSP47 regulates IRE1, and this interaction is a flexible and adaptive part of the UPR pathway, helping cells cope with ER stress.

#### 2.2.2 VEGFR2 signalling mediated angiogenesis

**FIGURE 3:**
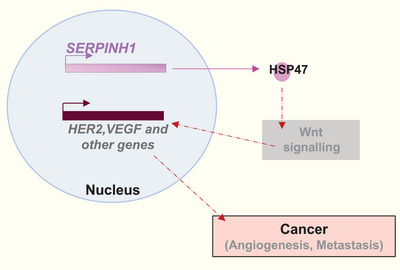
HSP47in cancer associated angiogenesis. It promotes angiogenesis via Wnt‐Vascular endothelial growth factor (VEGF)‐HSF‐1 signalling.^34^, ^66^

Vascular endothelial growth factor (VEGF) plays a focal role in the formation of blood vessels (vasculogenesis and angiogenesis).^31^ Excessive VEGF disrupts intracellular barriers, increases leakage of the choroid plexus endothelia, evokes oedema and activates the inflammatory pathway in disorders like cancer^32^ and cardiovascular diseases.^33^ In glioblastoma, silencing HSP47 showed a reduction in the VEGF, HIF1α, PLCγ, ERK1/2 and Src and angiogenic gene expressions. It had a vice versa effect on its up‐regulation, indicating a possible role of HSP47 in enhancing angiogenesis in glioma angiogenesis through the HIF1α‐VEGFR2 signalling^34^ (Figure 3).

In head‐and‐neck squamous carcinoma, there is an increase in both HSP47 and VEGF expression under hypoxia. However, when treated with a stress kinase inhibitor, this increase reversed.^35^ ERK pathway activation combined with C–C motif chemokine ligand 2 induction promotes HSP47‐induced angiogenesis in bladder cancer.^36^ Moreover, HSP47 inhibitors have also been shown to reduce VEGF‐induced fibrovascular retinal fibrosis.^37^ Therefore, these findings highlight the role of HSP47 in angiogenesis.

#### 2.2.3 Platelets and thrombosis

**FIGURE 4:**
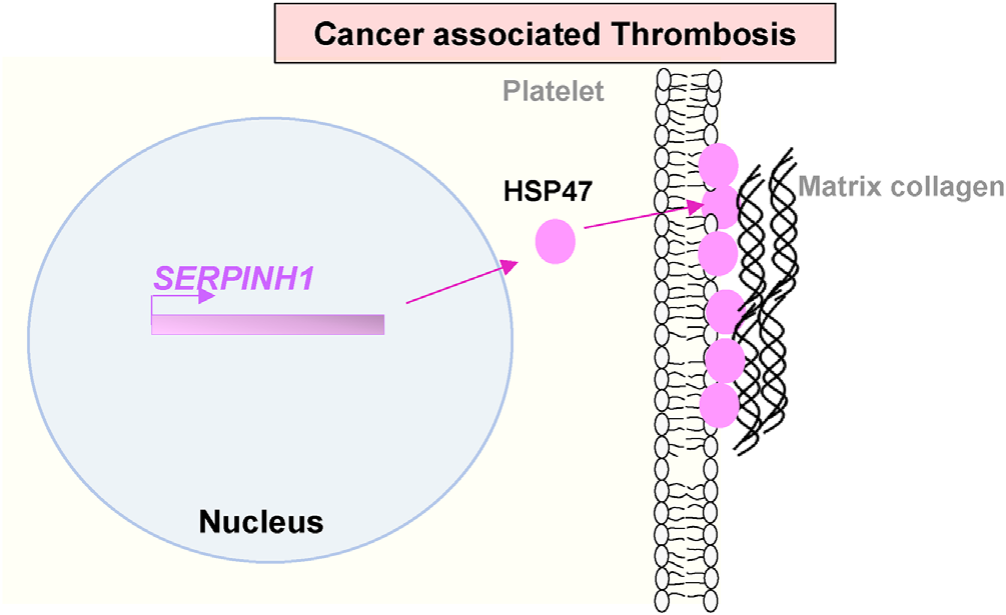
HSP47in cancer associated thrombosis. Cell membrane‐bound HSP47 helps platelets stick to the collagen matrix, promoting platelet aggregation and blood clotting in cancer.^40^, ^41^

Thrombosis refers to the process of blood clot formation. It is initiated by platelets adhering to the injury site and releasing molecules like thromboxane A2 and ADP which promotes platelet aggregation through fibrinogen and fibrin clot formation.^38^ Dysregulation of these processes contributes to pathologies like atherosclerosis, where platelet‐driven thrombosis can lead to serious cardiovascular events.^39^ Studies have revealed that HSP47 is exposed on the surface of activated human platelets.^40^ It has been identified to help platelet collagen‐binding to promote platelet aggregation, highlighting its significance in platelet function and coagulation processes41 (Figure 4). Interestingly, HSP47 has been shown to promote cancer metastasis by enhancing collagen‐dependent cancer cell‐platelet interaction.^8^ Therefore, HSP47 may be one of the important players in the platelet paradigm and its physiological processes.^42^

Altogether, Hsp47 demonstrates functions extending beyond collagen assembly, engaging in diverse cellular mechanisms. Nevertheless, the underlying mechanisms governing these functions remain to be fully elucidated.

## 3 HSP47 IN HUMAN DISEASES

Given the emerging functions of HSP47 in diverse biological processes, it is not surprising that its expression and abundance are increasingly linked to disorders, encompassing both collagenopathies (fibrosis^43^) and non‐collagenopathies (neurological disorders^11^). In the following, we present a few examples that document the broad spectrum of such disorders associated with HSP47.

### 3.1 Fibrosis

**FIGURE 5:**
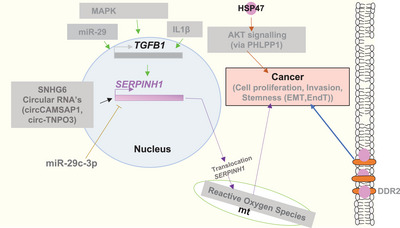
HSP47 linked to fibrosis and cancer stemness, proliferation and survival. HSP47 is up‐regulated in fibrosis and cancers through TGFβ1 signalling,^60^ circular RNAs^62^ or small nucleolar RNA Host Gene 6 (SNHG6)^61^ or cancer survival and metastasis (via AKT‐PHLPP1 signalling^65^). Translocation of the HSP47 gene causes mitochondrial (mt) stress and reactive oxygen species (ROS) to induce cancer.^67^ HSP47 bound discoidin domain receptor tyrosine kinase 2 (DDR2) on cell surface promotes cancer.^68^

Fibrosis is attributed to excess deposition of ECM components majorly collagen defined by the hardening, overgrowth and scarring of various tissues leading to organ dysfunction and eventual death.^44^ The severity of the disease varies depending on the type of tissue affected.^45^ Myofibroblasts, activated cells that produce aberrant collagen, are key mediators of fibrosis.^45^ They are generated through processes such as epithelial/endothelial‐mesenchymal transition (EMT/EndMT)^46^ and from circulating fibroblast‐like cells called fibrocytes, derived from bone marrow stem cells.^47^ A cycle of myofibroblast accumulation occurs due to their resistance to apoptosis, influencing EMT through increased matrix stiffness and positive feedback on TGF‐β1 activation.^44^ HSP47 expression is up‐regulated by unsolicited TGF‐β1 activation through the MAPK signalling pathway or by IL1β alone or in combination with TGF‐β^48^, ^49^ (Figure 5). Both mediators enhance HSF 1 trimerization, increasing its affinity for the heat shock element^48^ and leading to HSP47up‐regulation causing deposition of defective collagens in tissues, contributing to a fibrotic environment that further amplifies HSP47 up‐regulation.^43^ Clinical data shows that Hsp47 is up‐regulated in various tissue‐specific fibrosis and can act as a molecular signature to assess the disease progression.^50^, ^51^, ^52^, ^53^, ^54^, ^55^, ^56^ Elevated HSP47 in fibrosis is also associated with myocardial infarction,^57^ atherosclerotic arteries^57^ and scleroderma patients.^58^ In conclusion, HSP47 emerges as a critical player in fibrotic processes across diverse tissues, and its up‐regulation has a diagnostic potential for assessing disease progression.

### 3.2 Cancers

Fibrotic diseases and cancer share several characteristics; both pathologies are characterized by uncontrolled cell proliferation, genetic and cellular alterations, and tissue invasion. In the last decade, accumulating evidence suggests the role of HSP47 in the progression of various cancers. The *SERPINH1* (HSP47 gene) is located on chromosome 11q13, a region usually amplified in human cancers.^59^ Up‐regulation of HSP47 is associated with increased cancer progression and its expression in the tumour‐associated stroma.^12^ TGF‐β signalling appears to play a crucial role in regulating HSP47 expression to promote HSP47‐induced tumour progression and stemness in glioblastoma.^60^ Small nucleolar RNA host gene 6 up‐regulation has also been shown to activate HSP47 expression by competitive binding to miR‐139‐5p to promote hepatocellular carcinoma.^61^ Recently, circular RNA has been shown to enhance HSP47 expression by binding to its mRNA and promoting various cancer progressions. For instance, in nasopharyngeal carcinoma, splicing factor‐derived circular RNA (circCAMSAP1) has accelerated tumourigenesis via the HSP47/c‐Myc positive feedback loop.^62^ Another circular RNA, circ‐TNPO3, binds to Insulin‐like growth factor 2 mRNA‐binding protein 2, destabilizing HSP47 mRNA and inhibiting clear cell renal cell carcinoma metastasis.^63^ High levels of HSP47 have been linked to various cancer promoting cascades getting activated. For example, HSP47 promotes cell migration and invasion through the AKT signalling pathway in non‐small cell lung cancer.^64^ It has also been shown to promote the growth of colorectal cancer (CRC) tumours and suppress the efficacy of chemotherapy via modulation of AKT‐PHLPP1 signalling.^65^ It also regulates the EMT of gastric cancer metastasis through the Wnt/β‐catenin signalling.^66^ Additionally, translocation of HSP47 has shown the generation of mitochondrial reactive oxygen species in human neuroblastoma.^67^ Moreover, HSP47 sustains the membrane localization and stability of discoidin domain‐containing receptor 2, promoting EMT in breast cancer.^68^ Clinical data suggest that HSP47 has a role in the prognosis of laryngeal squamous cell carcinoma by inhibiting cell viability and invasion and promoting apoptosis.^69^ Preoperative HSP47 levels identify CRC patients with lymph node metastasis and poor prognosis.^12^ HSP47 is regulated by miR‐29 during breast cancer progression. The underlying possible mechanism is through the HSP47/Smad3 signalling pathway.^70^, ^71^, ^72^ Tumour‐suppressive microRNA‐29a inhibits invasion and cancer cell migration via targeting HSP47 in cervical squamous cell carcinoma.^73^ These findings highlight the multifaceted role of HSP47 in cancer and related pathological conditions (Figure 5), positioning it as a promising target for further research and potential therapeutic interventions.

### 3.3 Osteogenesis imperfecta

OI, also known as brittle bone disease, is a phenotypically and molecularly heterogeneous group of inherited skeletal disorders, which is characterized by irregular connective tissue morphology and mineralization.^74^, ^75^ OI can be broadly classified into two types. Dominant OI forms (90% of all cases) are generally induced by mutations of type I collagen itself, whereas recessive OI forms originate due to alterations in collagen posttranslational modification machinery.^76^ HSP47 deficiency in recessive OI fibroblasts has been shown to promote inadequate collagen deposition causing inefficiently mineralized bone fragility.^77^, ^78^ Homozygous missense mutation, point mutation or deletion in *SERPINH1* (c.233T > C, p.L78P,^79^ c.338_357del22,^80^ c.314_325del p,^81^ (c.149 T > G, p. L50R,c.1214 G > A, p. R405H^82^), p.(R222S)^83^) has been reported to cause HSP47 deficiency manifesting severe OI with blue sclerae and dentogenesis imperfect. Moreover, a functional single nucleotide polymorphism in the promoter of *SERPINH1* has been associated with increasing the risk for preterm rupture of membranes.^84^ OI patients carrying bi‐allelic variants of KDELR2 (KDEL ER protein retention receptor 2) were identified to impair the KDEL receptor binding to HSP47, hindering HSP47's dissociation from collagen and release into the ECM space.^85^ In summary, HSP47 mutation or deficiency impairs posttranslational modification, trafficking and deposition of ECM collagen in recessive OI patients.

### 3.4 Diabetes mellitus

Diabetes mellitus is a long‐term metabolic condition characterized by elevated blood glucose levels due to low insulin production or the body's inability to use insulin effectively.^86^ Type 1 diabetes mellitus is often classified as an autoimmune disorder.^87^ In this form of diabetes, the immune system mistakenly targets and destroys the insulin‐producing beta cells in the pancreas.^88^ The autoimmune attack leads to a significant reduction or complete absence of insulin production, resulting in elevated blood glucose levels.^87^, ^88^ The exact cause of this autoimmune response is not fully understood, but a combination of genetic and environmental factors is believed to play a role.^87^, ^88^, ^89^ In diabetic nephropathy, advanced glycation end products increase the expression of HSP47 in association with collagens through TGF‐β.^90^ Hsp47 up‐regulation in the later stages (sclerotic phase) of streptozotocin‐induced diabetic nephropathy is associated with glomerulosclerosis and tubulointerstitial fibrosis.^91^ High glucose in retinal Müller cells induces HSP47 up‐regulation and the secretion of inflammatory factors through the IRE1α/XBP1/HIF‐1α pathway in diabetic retinopathy.^91^ HSP47 also induces diabetes‐related kidney disease in children, positioning it as a potential target for diabetic therapeutic interventions.^92^ Therefore, HSP47 is an important chaperone that contributes to disease progression in diabetes.

### 3.5 Neurodegenerative diseases

**FIGURE 6:**
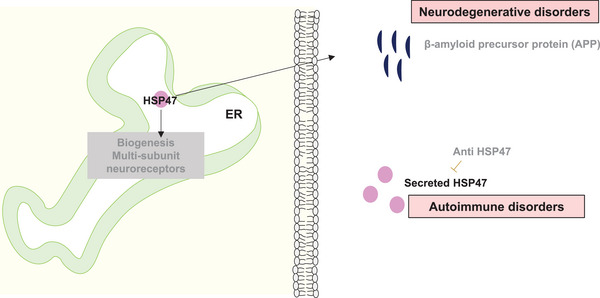
HSP47 in autoimmune and neurological disorders. HSP47 interacts with multi‐subunit neuroreceptors and promotes β amyloid precursor protein (APP) secretion in neurodegenerative diseases^11^, ^95^ and contributes to autoimmune disorders through unsolicited secretion.^97^

Neurodegenerative diseases refer to a group of disorders characterized by progressive degeneration of the nervous system.^93^ Common neurodegenerative diseases include Parkinson's disease, Alzheimer's disease (AD) and Huntington's disease.^94^ The exact causes of neurodegenerative diseases are complex and involve a combination of genetic, environmental and age‐related factors.^94^ Work by the group of Ferdinando Di Cunto revealed an association of HSP47 with the β‐amyloid precursor protein, a common component enriched in amyloid plaques primarily found in AD mouse models and patients.^11^ Inhibition of HSP47 reduced the levels of secreted Aβ peptides, implying HSP47 is a prominent target for preventing the formation and growth of amyloid plaques in AD patients. Moreover, HSP47 has been associated with the biogenesis of multi‐subunit neuroreceptors in the ER, indicating its role in neurological processes^95^ (Figure 6). These findings collectively emphasize the significance of HSP47 in neurological diseases and highlight its potential as a therapeutic target for conditions involving collagen synthesis and ECM remodelling.

### 3.6 Rheumatic autoimmune diseases

Autoimmune rheumatic diseases (ARDs) are primarily associated with the joints, bones, muscle and connective tissue defects that are challenging to diagnose during early stages, presenting nonspecific symptoms and signs.^96^ Sera of patients with ARDs such as rheumatoid arthritis (RA), Sjögren's syndrome, systemic lupus erythematosus and mixed connective tissue disease (MCTD) have significantly high amounts of HSP47 protein and autoantibody levels^97^ (Figure 6). Notably, the sera of MCTD patients have elevated levels of HSP47 antigen and anti‐HSP47 autoantibodies, which is considered a useful marker. In RA patients, up‐regulated levels of HSP47 in synovial fibroblasts have been reported as a reliable marker for synovial fibroblasts quantification.^98^

The above‐mentioned examples linking Hsp47 expression to various diseases highlight the significance of Hsp47 in diverse pathophysiology. Although Hsp47 may not be the cause for the progression of all the disease entities, it could perpetuate and potentially worsen the underlying pathophysiology. In either scenario, the prevalence and correlation of Hsp47 with diseases make it compelling for diagnostics.

## 4 Diagnostic potential of HSP47

**FIGURE 7:**
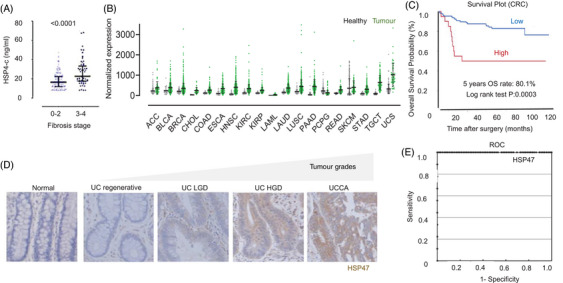
HSP47 as a potent biomarker. (A) HSP47‐c levels as a prognostic determinant for alcohol‐related liver disease patients divided into two cohorts based on histological fibrosis stage, F0‐2 and F3‐4.^100^ (B) HSP47 in human tumours (green) compared to their healthy counterparts (grey). (C) High‐level HSP47 (Red) is associated with lower survival probability in colorectal cancer.^12^ (D) HSP47 expression used for grading different ulcerative colitis (UC)‐associated carcinomas as a biomarker. UCLGD (UC low‐grade dysplasia); UCHGD (UC high‐grade dysplasia); UCCA (UC‐associated adenocarcinoma).^103^ (E) Highest diagnostic accuracy for HSP47 which discriminated between acute interstitial pneumonia (AIP) and the other IIP patients and healthy volunteers with 100% sensitivity, 98.5% specificity and a diagnostic accuracy of 98.7% assessed by measuring serum HSP47 level with area under the curve of 1.000.^105^ *Source*: All figure parts are reproduced with permission. (B) Data of patients obtained from https://oncodb.org/index.html.

HSP47 has garnered significant attention for its potential as a diagnostic biomarker across various diseases, particularly in collagen‐related disorders such as fibrosis, making it a promising diagnostic biomarker.^5^, ^16^ In Crohn's disease (CD), HSP47 has been shown to differentiate between fibrotic and non‐fibrotic forms, presenting itself as a prospective diagnostic marker for treating CD‐related fibrosis.^99^ Furthermore, serological assessment of HSP47 has been identified as a signature of the fibrosis stage in early compensated alcohol‐related liver disease (Figure 7A), emphasizing its diagnostic relevance.^100^ Additionally, HSP47 serves as a prognostic marker in various cancers as it is over‐expressed in most tumours (Figure 7B). In lung squamous cell carcinoma, it has been shown to distinguish between histopathological grades.^101^ In CRC, HSP47‐positive cells in the cancer stroma are proposed as a predictive biomarker for lymph node metastasis and poor prognosis^12^ (Figure 7C). In pancreatic ductal adenocarcinoma, HSP47 expression is almost universally intense in ductal adenocarcinoma‐associated stromal desmoplasia, underscoring its potential diagnostic implications.^102^In ulcerative colitis (UC), where there is an elevated risk of colorectal carcinoma, HSP47 overexpression stands out as a unique signature for grading different UC‐associated carcinomas^103^ (Figure 7D). Furthermore, the use of HSP47 as a marker for increased collagen metabolism has been instrumental in comparing different treatments for bladder cancer, shedding light on its potential utility in assessing treatment responses.^104^ Beyond cancers, patients diagnosed with acute interstitial pneumonia exhibit significantly elevated serum levels of HSP47 compared to those with cryptogenic organizing pneumonia (COP), nonspecific interstitial pneumonia, idiopathic pulmonary fibrosis and healthy volunteers. The diagnostic performance, evaluated using the ROC threshold, demonstrated exceptional sensitivity (100.0%), specificity (98.5%) and an overall diagnostic accuracy of 98.7%^105^ (Figure 7E). Furthermore, HSP47 expression, influenced by its intron or synonymous variants, is linked to increased body fat traits, determining the extent of body adiposity and suggesting its potential as a diagnostic marker in obesity.^106^ Taken together, HSP47 holds promise as a versatile biomarker and potential therapeutic target across a wide range of pathological conditions.^13^

## 5 TECHNIQUES USED FOR SCREENING HSP47

To date, a variety of techniques, including immunohistochemistry (IHC), Western blotting (WB),^17^ ELISA^105^ and q‐PCR,^10^ have been employed to study HSP47 and its associated processes (Table 1). Although each technique provides unique insights into HSP47 expression and function, not all are readily adaptable for clinical use. In a clinical context, IHC WB and ELISA are commonly used in research laboratories and could potentially be adapted for diagnostic purposes, provided standardized protocols and quality control measures are established. IHC allows for the visualization of HSP47 expression in tissue samples, offering insights into its localization within specific tissues with limited quantitative information.^6^ WB, on the other hand, facilitates the quantitative assessment of HSP47 protein levels in cell lysates, enabling the determination of HSP47 abundance but with Hsp47 antibody having high quality and specificity.^17^ Other approaches like fluorescence microscopy,^17^ flow cytometry^9^ and in situ hybridization^107^ offer valuable insights into HSP47's subcellular distribution, single‐cell expression and mRNA localization, and their clinical implementation may pose challenges due to the need for specialized equipment, cost and expertise.

**TABLE 1:** Techniques for diagnosing heat shock protein 47 (HSP47).

Diagnostic technique	Pros	Cons	Potential clinical benefits
**Immunohistochemistry** (IHC)^6^	Visualizes HSP47 expression in tissue samples	Limited quantitative data	Identifying HSP47 overexpression in specific tissues
**Western blotting** ^17^	Quantitative assessment of HSP47 protein levels	Requires high‐quality antibodies for specificity	Determining HSP47 abundance in various cell lysates
**ELISA** (enzyme‐linked immunosorbent assay)^108^	Allows quantitative measurement of HSP47 in biological fluids	Limited to extracellular or secreted HSP47	Assessing HSP47 levels in blood or urine for systemic indications
**qPCR** ^109^	Detects HSP47 mRNA levels, providing insight into transcriptional regulation	Limited information on posttranslational modifications	Examining gene expression changes in response to stimuli
**Fluorescence microscopy** ^17^	Enables visualization of HSP47 localization within cells	Limited resolution for certain cellular structures	Identifying subcellular localization and organelle interactions
**Flow cytometry** ^17^	Quantifies HSP47 expression at the single‐cell level	Requires dissociation of tissues for single‐cell analysis	Characterizing HSP47 levels in distinct cell populations
**In situ hybridization** (ISH)^107^	Visualizes the localization of HSP47 mRNA in tissues	Limited quantitative data	Correlating HSP47 expression with tissue‐specific pathology

ELISA shows promise for clinical use, allowing quantitative measurement of HSP47 in biological fluids, potentially indicating its presence systemically. From a diagnostic point of view, it is likely the preferred non‐invasive method for real‐time detection of HSP47 compared to biopsy tissues analysis. However, this technique is only limited to detecting ECM or secreted HSP47 unless cell lysates are used.^108^ q‐PCR, used to detect HSP47 mRNA levels, may be employed in a clinical setting for evaluating gene expression profiles associated with HSP47, particularly in diseases like cancer.^109^ Taken together, biochemical techniques for detecting HSP47 are available, but their clinical implementation requires careful optimization, protocol standardization and quality control measures for reliability. These techniques offer a comprehensive understanding of HSP47's functional importance and diagnostic potential in human disorders, potentially revealing insights into its role in pathologies affecting cells with limited regenerative potential. Given HSP47's involvement in various cellular processes like protein folding, cell membrane display, secretion and tissue homeostasis, perturbations in its biology could have detrimental effects. Further advancements in HSP47 detection technologies could offer new insights into its activities and consequences. Ideally, generalized guidelines are essential for their clinical applications. For instance, standardized ELISA and qPCR assays, with established normal ranges for HSP47 levels, distinguishing between ECM and intracellular isoforms of HSP47 with defined positive and negative controls is a necessity.

## 6 HSP47 IN THERAPY

HSP47 is an established therapeutic target for treating fibrosis and cancer.^110^, ^111^, ^112^ Here, we discuss a few examples of available therapeutic concepts for targeting HSP47 (Table 2). Small molecule inhibitors are one of the most accessible drug molecules that bind to proteins and interfere with their biological functions.^113^ Small‐molecule inhibitors targeting HSP47 have shown potential for controlling fibrosis and metastasis at low micromolar concentrations.^110^, ^112^ Using chemical inhibitors, it is possible to reduce the levels of secreted Aβ peptides by targeting HSP47 expression or interfering with its activity.^11^ HSP47 Inhibitor (Col003) reduced collagen‐induced platelet activation against brain damage induced by ischemic stroke.^111^ Pirfenidone, a commercially available drug, is effectively reduces murine bleomycin‐induced pulmonary fibrosis by attenuating HSP47 expression.^114^ Aspirin has shown a protective effect against renal damage under stress conditions by targeting HSP47‐mediated pathways in poultry.^115^, ^116^ SB203580 (4‐(4‐fluorophenyl)‐2‐(4‐methylsulfinylphenyl)‐5‐(4‐pyridyl)‐imidazole) is a stress kinase inhibitor that inhibits p38 MAPK by blocking MAPKAPK‐2 activation and HSP phosphorylation.^117^ Its treatment has shown down‐regulation of collagen XVIII, CBP2/Hsp47 and VEGF expression induced by liver fibrosis and hypoxia.^118^, ^119^ Another approach is to use peptide drugs that have shown great potential for targeting HSP47.120 Engineered LDS affinity peptide with lanthanide‐doped SPIO nanoparticles treatment has shown effective targeted therapy in cancer cells.^120^ Natural compounds and derivatives, identified through in silico methodology, have shown a potential to modulate HSP47. Compounds like silymarin and curcumin have been shown to block the HSP47‐procollagen complex, demonstrating therapeutic applicability in conditions such as liver fibrosis, keloids and pulmonary fibrosis.^121^, ^122^ Furthermore, calcium channel blockers, such as meloxicam, have demonstrated the potential to reduce fibrosis by down‐regulating the expression of HSP47 and collagen genes in animal models.^123^, ^124^ Tetrandrine treatment in BDL rats has shown a decrease in HSP47, collagen 1, α‐SMA and Pcol1A1 in fibrotic rat livers.^125^, ^126^ Receptor tyrosine kinase inhibitors like Nintedanib have also shown promise in down‐regulating the expression of HSP47 and collagen genes, offering potential therapeutic benefits in fibrotic diseases.^127^, ^128^ Moreover, vitamin A‐coupled liposomes containing HSP47 siRNA are effective in treating skin fibrosis in chronic graft‐versus‐host disease.^129^ ND‐L02‐s0201, an HSP47 siRNA lipid nanoparticle, has been shown to reverse interstitial pulmonary fibrosis in preclinical rat models.^130^ HSP47 siRNA with NOX4‐modulating mesoporous silica‐based nanoparticles has shown promising dermal delivery for treating fibrosis.^130^ Using a preclinical model, an RNA ligand‐tethered lipid nanoparticle (AA‐T3A‐C12) has shown about ∼65% silencing of HSP47, leading to a significant reduction in liver fibrosis.^131^ In 2022, a Phase 2 trial investigated the efficacy of BMS‐986263, aimed at reducing hepatic fibrosis in HCV‐SVR patients by targeting HSP47 mRNA.^132^, ^133^ Although there were slight improvements in METAVIR and Ishak scores at Week 12, further research is necessary to fully understand its potential benefits, despite the generally manageable adverse events reported.

**TABLE 2:** Therapeutic strategies used to target heat shock protein 47 (HSP47).

Therapeutic approach	Pros	Cons	Potential clinical benefits	Phase
**Small‐molecule inhibitors** ^110^, ^111^, ^112^, ^114^	Broad applicability with potential for oral administration	May have off‐target effects on other cellular processes	Inhibiting HSP47 function in various fibrotic diseases	Preclinical trail
**Peptide‐based inhibition** ^120^	Targeted disruption of HSP47‐procollagen interaction	Limited tissue penetration for certain peptides	Blocking collagen secretion and fibrosis progression	
**Natural compounds and derivatives** ^121^, ^122^	Potential therapeutic agents with diverse mechanisms	Variable bioavailability and efficacy among compounds	Modulating HSP47 levels using natural anti‐fibrotic agents	
**Calcium channel blockers** ^123^, ^124^	Impact on HSP47 expression through alternative pathways	Systemic effects on calcium homeostasis and other processes	Regulating fibrosis by modulating calcium signalling	
**Receptor antagonists (Nintedanib)** ^127^, ^128^	Inhibition of crucial signalling pathways involved in fibrosis	Side effects and tolerability concerns in some patients	Slowing disease progression in conditions like IPF	
**siRNA targeting** ^131^	Specific suppression of HSP47 expression	Delivery challenges for effective siRNA uptake	Attenuating fibrosis by reducing collagen synthesis	BMS‐986263, Phase2 clinical trail(2022) (NCT03420768)^132^

Considering these broad spectra of therapeutic approaches targeting HSP47, it is evident that there is a rich landscape of potential treatments for fibrosis and cancer. However, there is still much to be explored. Future research should focus on optimizing existing therapies, discovering new compounds and investigating combination therapies to enhance the efficacy of HSP47‐targeting treatments while minimizing adverse effects. Furthermore, although HSP70, HSP90 and HSP47 have received significant attention,^110^, ^134^ exploring other HSP in clinical trials could provide valuable insights into accelerating the development of effective treatments for numerous disease entities.^135^

## 7 CONCLUSION

With the advent of new functions of HSP47, it is no surprise that this chaperone protein is implicated in various diseases, including fibrosis, cancer and neurodegenerative disorders accentuating its significance as a diagnostic biomarker and therapeutic target. Its dynamic expression patterns serve as an indicator of disease progression and therapeutic response. Targeting HSP47 offers promising interventions, particularly in conditions involving excessive collagen deposition and fibrosis. Advanced detection techniques like immunoassays have enabled precise quantification and localization of HSP47 that can guide personalized treatment strategies. Overall, exploiting HSP47's multifunctional roles holds promise for improving clinical management and developing therapeutics for enhanced healthcare.

## AUTHOR CONTRIBUTIONS

**Essak. S. Khan**: Conceptualization; literature survey; figures preparation; writing original draft; review and editing. **Tobias Däinghaus**: Literature Survey; writing original draft and editing.

## CONFLICT OF INTEREST STATEMENT

The authors declare no conflicts of interest.

## ETHICS APPROVAL AND CONSENT TO PARTICIPATE

Not applicable.

## Data Availability

Not applicable.

## References

[1] Kim YE , Hipp MS , Bracher A , Hayer‐Hartl M , Ulrich Hartl F . Molecular chaperone functions in protein folding and proteostasis. Annu Rev Biochem. 2013;82:323‐355. 23746257 10.1146/annurev-biochem-060208-092442

[2] Barral JM , Broadley SA , Schaffar G , Hartl FU . Roles of molecular chaperones in protein misfolding diseases. Semin Cell Dev Biol. 2004;15:17‐29. 15036203 10.1016/j.semcdb.2003.12.010

[3] Ishida Y , Nagata K . Hsp47 as a collagen‐specific molecular chaperone. Methods Enzymol. 2011;499:167‐182. 21683254 10.1016/B978-0-12-386471-0.00009-2

[4] Hendershot LM , Bulleid NJ . Protein‐specific chaperones: the role of hsp47 begins to gel. Curr Biol. 2000;10:R912‐915. 11137028 10.1016/s0960-9822(00)00850-2

[5] Omari S , Makareeva E , Gorrell L , Jarnik M , Lippincott‐Schwartz J , Leikin S . Mechanisms of procollagen and HSP47 sorting during ER‐to‐Golgi trafficking. Matrix Biol. 2020;93:79‐94. 32562852 10.1016/j.matbio.2020.06.002 PMC8932071

[6] Brown KE , Broadhurst KA , Mathahs MM , Brunt EM , Schmidt WN . Expression of HSP47, a collagen‐specific chaperone, in normal and diseased human liver. Lab Invest. 2005;85:789‐797. 15806139 10.1038/labinvest.3700271

[7] Sepulveda D , Rojas‐Rivera D , Rodríguez DA , et al. Interactome screening identifies the ER luminal chaperone Hsp47 as a regulator of the unfolded protein response transducer IRE1α. Mol Cell. 2018;69:238‐252. e237. 29351844 10.1016/j.molcel.2017.12.028

[8] Xiong G , Chen J , Zhang G , et al. Hsp47 promotes cancer metastasis by enhancing collagen‐dependent cancer cell‐platelet interaction. Proc Natl Acad Sci. 2020;117:3748‐3758. 32015106 10.1073/pnas.1911951117 PMC7035603

[9] Lakos G , Takagawa S , Chen S‐J , et al. Targeted disruption of TGF‐beta/Smad3 signaling modulates skin fibrosis in a mouse model of scleroderma. Am J Pathol. 2004;165:203‐217. 15215176 10.1016/s0002-9440(10)63289-0 PMC1618525

[10] Razzaque MS , Kumatori A , Harada T , Taguchi T . Coexpression of collagens and collagen‐binding heat shock protein 47 in human diabetic nephropathy and IgA nephropathy. Nephron. 1998;80:434‐443. 9832643 10.1159/000045217

[11] Bianchi FT , Camera P , Ala U , et al. The collagen chaperone HSP47 is a new interactor of APP that affects the levels of extracellular beta‐amyloid peptides. PLoS ONE. 2011;6:e22370. 21829458 10.1371/journal.pone.0022370 PMC3145648

[12] Mori K , Toiyama Y , Okugawa Y , et al. Preoperative heat shock protein 47 levels identify colorectal cancer patients with lymph node metastasis and poor prognosis. Oncol Lett. 2020;20:333. 33123244 10.3892/ol.2020.12196 PMC7583735

[13] Sauk JJ , Nikitakis N , Siavash H . Hsp47 a novel collagen binding serpin chaperone, autoantigen and therapeutic target. Front Biosci. 2005;10:107‐118. 15574354 10.2741/1513

[14] Taguchi T , Razzaque MS . The collagen‐specific molecular chaperone HSP47: is there a role in fibrosis? Trends Mol Med. 2007;13:45‐53. 17169614 10.1016/j.molmed.2006.12.001

[15] Verrico AK , Haylett AK , Moore JV . In vivo expression of the collagen‐related heat shock protein HSP47, following hyperthermia or photodynamic therapy. Lasers Med Sci. 2001;16:192‐198. 11482817 10.1007/pl00011354

[16] Ito S , Nagata K . Roles of the endoplasmic reticulum‐resident, collagen‐specific molecular chaperone Hsp47 in vertebrate cells and human disease. J Biol Chem. 2019;294:2133‐2141. 30541925 10.1074/jbc.TM118.002812 PMC6369284

[17] Ono T , Miyazaki T , Ishida Y , Uehata M , Nagata K . Direct in vitro and in vivo evidence for interaction between Hsp47 protein and collagen triple helix. J Biol Chem. 2012;287:6810‐6818. 22235129 10.1074/jbc.M111.280248 PMC3307285

[18] Köhler A , Mörgelin M , Gebauer JM , et al. New specific HSP47 functions in collagen subfamily chaperoning. FASEB J. 2020;34:12040‐12052. 32716577 10.1096/fj.202000570R

[19] Forrester A , De Leonibus C , Grumati P , et al. A selective ER‐phagy exerts procollagen quality control via a Calnexin‐FAM134B complex. EMBO J. 2019;38:e99847. 30559329 10.15252/embj.201899847 PMC6331724

[20] Ishida Y , Yamamoto A , Kitamura A , et al. Autophagic elimination of misfolded procollagen aggregates in the endoplasmic reticulum as a means of cell protection. Mol Biol Cell. 2009;20:2744‐2754. 19357194 10.1091/mbc.E08-11-1092 PMC2688553

[21] Ishida Y , Kubota H , Yamamoto A , Kitamura A , Bächinger HP , Nagata K . Type I collagen in Hsp47‐null cells is aggregated in endoplasmic reticulum and deficient in N‐propeptide processing and fibrillogenesis. Mol Biol Cell. 2006;17:2346‐2355. 16525016 10.1091/mbc.E05-11-1065 PMC1446091

[22] Ishikawa Y , Bächinger HP . A molecular ensemble in the rER for procollagen maturation. Biochim Biophys Acta. 2013;1833:2479‐2491. 23602968 10.1016/j.bbamcr.2013.04.008

[23] Duran I , Nevarez L , Sarukhanov A , et al. HSP47 and FKBP65 cooperate in the synthesis of type I procollagen. Hum Mol Genet. 2015;24:1918‐1928. 25510505 10.1093/hmg/ddu608 PMC4355024

[24] Duran I , Martin JH , Weis MA , et al. A chaperone complex formed by HSP47, FKBP65, and BiP modulates telopeptide lysyl hydroxylation of type I procollagen. J Bone Miner Res. 2017;32:1309‐1319. 28177155 10.1002/jbmr.3095 PMC5466459

[25] Raote I , Ortega‐Bellido M , Santos AJ , et al. TANGO1 builds a machine for collagen export by recruiting and spatially organizing COPII, tethers and membranes. eLife. 2018;7:e32723. 29513218 10.7554/eLife.32723 PMC5851698

[26] Ishikawa Y , Ito S , Nagata K , Sakai LY , Bächinger HP . Intracellular mechanisms of molecular recognition and sorting for transport of large extracellular matrix molecules. Proc Natl Acad Sci USA. 2016;113:E6036‐E6044. 27679847 10.1073/pnas.1609571113 PMC5068301

[27] Walter P , Ron D . The unfolded protein response: from stress pathway to homeostatic regulation. Science. 2011;334:1081‐1086. 22116877 10.1126/science.1209038

[28] Mori K . Signalling pathways in the unfolded protein response: development from yeast to mammals. J Biochem. 2009;146:743‐750. 19861400 10.1093/jb/mvp166

[29] Lamriben L , Hebert DN . Activating and repressing IRE1α: the Hsp47 and BiP tug of war. Mol Cell. 2018;69:159‐160. 29351839 10.1016/j.molcel.2017.12.032 PMC6816461

[30] Yoneda A , Minomi K , Tamura Y . Heat shock protein 47 confers chemoresistance on pancreatic cancer cells by interacting with calreticulin and IRE1α. Cancer Sci. 2021;112:2803‐2820. 34109710 10.1111/cas.14976 PMC8253297

[31] Shim J , Madsen J . VEGF signaling in neurological disorders. Int J Mol Sci. 2018;19:275. 29342116 10.3390/ijms19010275 PMC5796221

[32] Stacker SA , Achen MG . The VEGF signaling pathway in cancer: the road ahead. Chin J Cancer. 2013;32:297‐302. 23419196 10.5732/cjc.012.10319 PMC3845619

[33] Touyz RM , Herrmann J . Cardiotoxicity with vascular endothelial growth factor inhibitor therapy. NPJ Precis Oncol. 2018;2:13. 30202791 10.1038/s41698-018-0056-z PMC5988734

[34] Wu ZB , Cai L , Lin SJ , et al. Heat shock protein 47 promotes glioma angiogenesis. Brain Pathol. 2016;26:31‐42. 25758142 10.1111/bpa.12256 PMC8029092

[35] Stewart J , Siavash H , Hebert C , Norris K , Nikitakis NG , Sauk JJ . Phenotypic switching of VEGF and collagen XVIII during hypoxia in head and neck squamous carcinoma cells. Oral Oncol. 2003;39:862‐869. 13679210 10.1016/s1368-8375(03)00110-6

[36] Ma W , Ou T , Cui X , et al. HSP47 contributes to angiogenesis by induction of CCL2 in bladder cancer. Cell Signal. 2021;85:110044. 34000383 10.1016/j.cellsig.2021.110044

[37] Recchia FM , Xu L . Differential expression of the collagen–binding protein Hsp47 in experimental retinal neovascularization. Invest Opht Vis Sci. 2005;46:3162‐3162.

[38] Koupenova M , Kehrel BE , Corkrey HA , Freedman JE . Thrombosis and platelets: an update. Eur Heart J. 2016;38:785‐791. 10.1093/eurheartj/ehw550 PMC11110018 28039338

[39] Wagner DD , Burger PC . Platelets in inflammation and thrombosis. Arterioscler Thromb Vasc Biol. 2003;23:2131‐2137. 14500287 10.1161/01.ATV.0000095974.95122.EC

[40] Kaiser WJ , Holbrook L‐M , Tucker KL , Stanley RG , Gibbins JM . A functional proteomic method for the enrichment of peripheral membrane proteins reveals the collagen binding protein Hsp47 is exposed on the surface of activated human platelets. J Proteome Res. 2009;8:2903‐2914. 19341245 10.1021/pr900027j

[41] Sasikumar P , Alouda KS , Kaiser WJ , et al. The chaperone protein HSP47: a platelet collagen binding protein that contributes to thrombosis and hemostasis. J Thromb Haemost. 2018;16:946‐959. 29512284 10.1111/jth.13998 PMC6434988

[42] Mcnicol A , Israels S . Beyond hemostasis: the role of platelets in inflammation, malignancy and infection. Cardiovasc Hematol Disord Drug Targets. 2008;8:99‐117. 18537597 10.2174/187152908784533739

[43] Yuan Y , Li N , Zeng L , Shen Z , Jiang C . Pathogenesis investigation of miR‐199‐5p in oral submucous fibrosis based on bioinformatics analysis. Oral Dis. 2019;25:456‐465. 30485610 10.1111/odi.13008

[44] Bellaye P‐S , Burgy O , Bonniaud P , Kolb M . HSP47: a potential target for fibrotic diseases and implications for therapy. Expert Opin Ther Targets. 2021;25:49‐62. 33287600 10.1080/14728222.2021.1861249

[45] Wynn T . Cellular and molecular mechanisms of fibrosis. J Pathol. 2008;214:199‐210. 18161745 10.1002/path.2277 PMC2693329

[46] Carew RM , Wang B , Kantharidis P . The role of EMT in renal fibrosis. Cell Tissue Res. 2012;347:103‐116. 21845400 10.1007/s00441-011-1227-1

[47] Kisseleva T , Brenner DA . Mechanisms of fibrogenesis. Exp Biol Med. 2008;233:109‐122. 10.3181/0707-MR-190 18222966

[48] Sasaki H , Sato T , Yamauchi N , et al. Induction of heat shock protein 47 synthesis by TGF‐β and IL‐1β via enhancement of the heat shock element binding activity of heat shock transcription factor 1. J Immunol. 2002;168:5178‐5183. 11994473 10.4049/jimmunol.168.10.5178

[49] Xiao H‐B , Liu R‐H , Ling G‐H , et al. HSP47 regulates ECM accumulation in renal proximal tubular cells induced by TGF‐β1 through ERK1/2 and JNK MAPK pathways. Am J Physiol Renal Physiol. 2012;303:F757‐F765. 22718885 10.1152/ajprenal.00470.2011 PMC3468491

[50] Razzaque MS , Ahmed AR . Collagens, collagen‐binding heat shock protein 47 and transforming growth factor‐β1 are induced in cicatricial pemphigoid: possible role (s) in dermal fibrosis. Cytokine. 2002;17:311‐316. 12061838 10.1006/cyto.2002.1020

[51] Naitoh M , Hosokawa N , Kubota H , et al. Upregulation of HSP47 and collagen type III in the dermal fibrotic disease, keloid. Biochem Biophys Res Commun. 2001;280:1316‐1322. 11162672 10.1006/bbrc.2001.4257

[52] Li L , Wu T , Huang J , et al. Expression of heat shock protein 47, transforming growth factor‐beta 1, and connective tissue growth factor in liver tissue of patients with Schistosoma japonicum‐induced hepatic fibrosis. Parasitology. 2015;142:341. 25111595 10.1017/S0031182014001115

[53] Ohba K , Miyata Y , Koga S , et al. Interstitial expression of heat‐shock protein 47 correlates with capillary deposition of complement split product C4d in chronic allograft nephropathy. Clin Transplant. 2005;19:810‐816. 16313330 10.1111/j.1399-0012.2005.00426.x

[54] Kamikawaji K , Seki N , Watanabe M , et al. Regulation of LOXL2 and SERPINH1 by antitumor microRNA‐29a in lung cancer with idiopathic pulmonary fibrosis. J Hum Genet. 2016;61:985‐993. 27488440 10.1038/jhg.2016.99

[55] Gawron K , Ochała‐Kłos A , Nowakowska Z , et al. TIMP‐1 association with collagen type I overproduction in hereditary gingival fibromatosis. Oral Dis. 2018;24:1581‐1590. 29989318 10.1111/odi.12938

[56] Razzaque MS , Taguchi T . Collagen‐binding heat shock protein (HSP) 47 expression in anti‐thymocyte serum (ATS)‐induced glomerulonephritis. J Pathol. 1997;183:24‐29. 9370943 10.1002/(SICI)1096-9896(199709)183:1<24::AID-PATH1106>3.0.CO;2-B

[57] Hagiwara S , Iwasaka H , Shingu C , Matumoto S , Hasegawa A , Noguchi T . Heat shock protein 47 (HSP47) antisense oligonucleotides reduce cardiac remodeling and improve cardiac function in a rat model of myocardial infarction. Thorac Cardiovasc Surg. 2011;59:386‐392. 21412710 10.1055/s-0030-1250658

[58] Chu H , Wu T , Wu W , et al. Involvement of collagen‐binding heat shock protein 47 in scleroderma‐associated fibrosis. Protein Cell. 2015;6:589‐598. 26091621 10.1007/s13238-015-0171-3 PMC4506285

[59] Schwab M . Amplification of oncogenes in human cancer cells. Bioessays. 1998;20:473‐479. 9699459 10.1002/(SICI)1521-1878(199806)20:6<473::AID-BIES5>3.0.CO;2-N

[60] Jiang X , Zhou T , Wang Z , Qi B , Xia H . HSP47 promotes glioblastoma stemlike cell survival by modulating tumor microenvironment extracellular matrix through TGF‐β pathway. ACS Chem Neurosci. 2017;8:128‐134. 27696866 10.1021/acschemneuro.6b00253

[61] Wu G , Ju X , Wang Y , Li Z , Gan X . Up‐regulation of SNHG6 activates SERPINH1 expression by competitive binding to miR‐139‐5p to promote hepatocellular carcinoma progression. Cell Cycle. 2019;18:1849‐1867. 31258024 10.1080/15384101.2019.1629772 PMC6681790

[62] Wang Y , Yan Q , Mo Y , et al. Splicing factor derived circular RNA circCAMSAP1 accelerates nasopharyngeal carcinoma tumorigenesis via a SERPINH1/c‐Myc positive feedback loop. Mol Cancer. 2022;21:62. 35227262 10.1186/s12943-022-01502-2 PMC8883650

[63] Pan X , Huang B , Ma Q , et al. Circular RNA circ‐TNPO3 inhibits clear cell renal cell carcinoma metastasis by binding to IGF2BP2 and destabilizing SERPINH1 mRNA. Clin Transl Med. 2022;12:e994. 35876041 10.1002/ctm2.994 PMC9309750

[64] Wu W , Hu Z , Xiong L , Zou J . Heat shock protein 47 promotes cell migration and invasion through AKT signal in non‐small cell lung cancer. Anticancer Drugs. 2022;33:268‐277. 34751174 10.1097/CAD.0000000000001262

[65] Chern Y , Zhang P , Ju H , T Tai I . Heat shock protein 47 promotes tumor survival and therapy resistance by modulating AKT signaling via PHLPP1 in colorectal cancer. Cancer Biol Med. 2020;17:343‐356. 32587773 10.20892/j.issn.2095-3941.2019.0261 PMC7309463

[66] Tian S , Peng P , Li J , et al. SERPINH1 regulates EMT and gastric cancer metastasis via the Wnt/β‐catenin signaling pathway. Aging (Albany NY). 2020;12:3574‐3593. 32091407 10.18632/aging.102831 PMC7066881

[67] Indo HP , Ito H , Nakagawa K , Chaiswing L , Majima HJ . Translocation of HSP47 and generation of mitochondrial reactive oxygen species in human neuroblastoma SK‐N‐SH cells following electron and X‐ray irradiation. Arch Biochem Biophys. 2021;703:108853. 33811847 10.1016/j.abb.2021.108853

[68] Chen J , Wang S , Zhang Z , Richards CI , Xu R . Heat shock protein 47 (HSP47) binds to discoidin domain‐containing receptor 2 (DDR2) and regulates its protein stability. J Biol Chem. 2019;294:16846‐16854. 31570520 10.1074/jbc.RA119.009312 PMC6851340

[69] Song X , Liao Z , Zhou C , et al. HSP47 is associated with the prognosis of laryngeal squamous cell carcinoma by inhibiting cell viability and invasion and promoting apoptosis. Oncol Rep. 2017;38:2444‐2452. 28849239 10.3892/or.2017.5893

[70] Tang X , Liu L , Liu S , Song S , Li H . MicroRNA‐29a inhibits collagen expression and induces apoptosis in human fetal scleral fibroblasts by targeting the Hsp47/Smad3 signaling pathway. Exp Eye Res. 2022;225:109275. 36206860 10.1016/j.exer.2022.109275

[71] Yamada Y , Sugawara S , Arai T , et al. Molecular pathogenesis of renal cell carcinoma: impact of the anti‐tumor miR‐29 family on gene regulation. Int J Urol. 2018;25:953‐965. 30153702 10.1111/iju.13783

[72] Xu R . MiR‐29/Hsp47 in ECM network. Oncoscience. 2015;2:843‐844. 26682273 10.18632/oncoscience.227 PMC4671948

[73] Yamamoto N , Kinoshita T , Nohata N , et al. Tumor‐suppressive microRNA‐29a inhibits cancer cell migration and invasion via targeting HSP47 in cervical squamous cell carcinoma. Int J Oncol. 2013;43:1855‐1863. 24141696 10.3892/ijo.2013.2145 PMC3834344

[74] Van Dijk FS , Sillence DO . Osteogenesis imperfecta: clinical diagnosis, nomenclature and severity assessment. Am J Med Genet A. 2014;164:1470‐1481. 10.1002/ajmg.a.36545 PMC4314691 24715559

[75] Jovanovic M , Guterman‐Ram G , Marini JC . Osteogenesis imperfecta: mechanisms and signaling pathways connecting classical and rare OI types. Endocr Rev. 2021;43:61‐90. 10.1210/endrev/bnab017 PMC8755987 34007986

[76] Forlino A , Marini JC . Osteogenesis imperfecta. Lancet North Am Ed. 2016;387:1657‐1671. 10.1016/S0140-6736(15)00728-X PMC7384887 26542481

[77] Parveen A , Kumar R , Tandon R , Khurana S , Goswami C , Kumar A . Mutational hotspots of HSP47 and its potential role in cancer and bone‐disorders. Genomics. 2020;112:552‐566. 30986427 10.1016/j.ygeno.2019.04.007

[78] Ishida Y , Kubota H , Yamamoto A , Kitamura A , Bächinger HP , Nagata K . Type I collagen in Hsp47‐null cells is aggregated in endoplasmic reticulum and deficient in N‐propeptide processing and fibrillogenesis. Mol Biol Cell. 2006;17:2346‐2355. 16525016 10.1091/mbc.E05-11-1065 PMC1446091

[79] Christiansen HE , Schwarze U , Pyott SM , et al. Homozygosity for a missense mutation in SERPINH1, which encodes the collagen chaperone protein HSP47, results in severe recessive osteogenesis imperfecta. Am Hum Genet. 2010;86:389‐398. 10.1016/j.ajhg.2010.01.034 PMC2833387 20188343

[80] Marshall C , Lopez J , Crookes L , Pollitt RC , Balasubramanian M . A novel homozygous variant in SERPINH1 associated with a severe, lethal presentation of osteogenesis imperfecta with hydranencephaly. Gene. 2016;595:49‐52. 27677223 10.1016/j.gene.2016.09.035

[81] Duran I , Nevarez L , Sarukhanov A , et al. HSP47 and FKBP65 cooperate in the synthesis of type I procollagen. Hum Mol Genet. 2014;24:1918‐1928. 25510505 10.1093/hmg/ddu608 PMC4355024

[82] Song Y , Zhao D , Xu X , et al. Novel compound heterozygous mutations in SERPINH1 cause rare autosomal recessive osteogenesis imperfecta type X. Osteoporos Int. 2018;29:1389‐1396. 29520608 10.1007/s00198-018-4448-2

[83] Syx D , Ishikawa Y , Gebauer J , et al. Aberrant binding of mutant HSP47 affects posttranslational modification of type I collagen and leads to osteogenesis imperfecta. PLoS Genet. 2021;17:e1009339. 33524049 10.1371/journal.pgen.1009339 PMC7877763

[84] Wang H , Parry S , Macones G , et al. A functional SNP in the promoter of the SERPINH1 gene increases risk of preterm premature rupture of membranes in African Americans. Proc Natl Acad Sci USA. 2006;103:13463‐13467. 16938879 10.1073/pnas.0603676103 PMC1557384

[85] Van Dijk FS , Semler O , Etich J , et al. Interaction between KDELR2 and HSP47 as a key determinant in osteogenesis imperfecta caused by Bi‐allelic variants in KDELR2. Am Hum Genet. 2020;107:989‐999. 10.1016/j.ajhg.2020.09.009 PMC7675035 33053334

[86] Diagnosis and classification of diabetes mellitus. Diabetes Care. 2009;32(Suppl 1):S62‐S67. 19118289 10.2337/dc09-S062 PMC2613584

[87] Kawasaki E . Type 1 diabetes and autoimmunity. Clin Pediatr Endocrinol. 2014;23:99‐105. 25374439 10.1297/cpe.23.99 PMC4219937

[88] Popoviciu MS , Kaka N , Sethi Y , Patel N , Chopra H , Cavalu S . Type 1 diabetes mellitus and autoimmune diseases: a critical review of the association and the application of personalized medicine. J Pers Med. 2023;13:422. 36983604 10.3390/jpm13030422 PMC10056161

[89] Roep BO , Thomaidou S , Van Tienhoven R , Zaldumbide A . Type 1 diabetes mellitus as a disease of the β‐cell (do not blame the immune system?). Nat Rev Endocrinol. 2021;17:150‐161. 33293704 10.1038/s41574-020-00443-4 PMC7722981

[90] Ohashi S , Abe H , Takahashi T , et al. Advanced glycation end products increase collagen‐specific chaperone protein in mouse diabetic nephropathy. J Biol Chem. 2004;279:19816‐19823. 15004023 10.1074/jbc.M310428200

[91] Sun X , Chen C , Liu H , Tang S . High glucose induces HSP47 expression and promotes the secretion of inflammatory factors through the IRE1α/XBP1/HIF‐1α pathway in retinal Müller cells. Exp Ther Med. 2021;22:1411. 34676004 10.3892/etm.2021.10847 PMC8524763

[92] Yildirim ZNY , Usta Akgul S , Alpay H , et al. PROGRESS STUDY: progression of chronic kidney disease in children and heat shock proteins. Cell Stress Chaperones. 2021;26:973‐987. 34671941 10.1007/s12192-021-01239-9 PMC8578260

[93] Gao H‐M , Hong J‐S . Why neurodegenerative diseases are progressive: uncontrolled inflammation drives disease progression. Trends Immunol. 2008;29:357‐365. 18599350 10.1016/j.it.2008.05.002 PMC4794280

[94] Ciurea AV , Mohan AG , Covache‐Busuioc RA , et al. Unraveling molecular and genetic insights into neurodegenerative diseases: advances in understanding Alzheimer's, Parkinson's, and Huntington's diseases and amyotrophic lateral sclerosis. Int J Mol Sci. 2023;24:10809. 37445986 10.3390/ijms241310809 PMC10341997

[95] Wang Y‐J , Di XJ , Han DY , et al. Hsp47 promotes biogenesis of multi‐subunit neuroreceptors in the endoplasmic reticulum. Biorxiv. 2022. 2022.2010.2024.513629. doi:10.1101/2022.10.24.513629 PMC11257679 38963323

[96] Safary A , Esalatmanesh K , Eftekharsadat AT , Jafari Nakjavani M‐R , Khabbazi A . Autoimmune inflammatory rheumatic diseases post‐COVID‐19 vaccination. Int Immunopharmacol. 2022;110:109061. 35978510 10.1016/j.intimp.2022.109061 PMC9283674

[97] Yokota S‐I , Kubota H , Matsuoka Y , et al. Prevalence of HSP47 antigen and autoantibodies to HSP47 in the sera of patients with mixed connective tissue disease. Biochem Biophys Res Commun. 2003;303:413‐418. 12659832 10.1016/s0006-291x(03)00352-8

[98] Izquierdo E , Cañete JD , Celis R , et al. Synovial fibroblast hyperplasia in rheumatoid arthritis: clinicopathologic correlations and partial reversal by anti‐tumor necrosis factor therapy. Arthritis Rheum. 2011;63:2575‐2583. 21547893 10.1002/art.30433

[99] Kurumi H , Takata T , Kanda T , et al. Investigating the role of heat shock protein 47 in fibrosis in Crohn's disease. Sci Rep. 2022;12:10966. 35768471 10.1038/s41598-022-15153-2 PMC9243024

[100] Lønsmann I , Gudmann NS , Manon‐Jensen T , et al. Serologically assessed heat shock protein 47 is related to fibrosis stage in early compensated alcohol‐related liver disease. Clin Biochem. 2022;104:36‐43. 34929150 10.1016/j.clinbiochem.2021.12.008

[101] Poschmann G , Sitek B , Sipos B , et al. Identification of proteomic differences between squamous cell carcinoma of the lung and bronchial epithelium. Mol Cell Proteomics. 2009;8:1105‐1116. 19176476 10.1074/mcp.M800422-MCP200 PMC2689775

[102] Maitra A , Iacobuzio‐Donahue C , Rahman A , et al. Immunohistochemical validation of a novel epithelial and a novel stromal marker of pancreatic ductal adenocarcinoma identified by global expression microarrays: sea urchin fascin homolog and heat shock protein 47. Am J Clin Pathol. 2002;118:52‐59. 12109856 10.1309/3PAM-P5WL-2LV0-R4EG

[103] Araki K , Mikami T , Yoshida T , et al. High expression of HSP47 in ulcerative colitis‐associated carcinomas: proteomic approach. Br J Cancer. 2009;101:492‐497. 19603022 10.1038/sj.bjc.6605163 PMC2720226

[104] Shackley DC , Haylett A , Whitehurst C , et al. Comparison of the cellular molecular stress responses after treatments used in bladder cancer. BJU Int. 2002;90:924‐932. 12460358 10.1046/j.1464-410x.2002.03024.x

[105] Kakugawa T , Yokota S‐I , Ishimatsu Y , et al. Serum heat shock protein 47 levels are elevated in acute interstitial pneumonia. BMC Pulm Med. 2014;14:48. 24650086 10.1186/1471-2466-14-48 PMC3994423

[106] Shin J , Toyoda S , Okuno Y , et al. HSP47 levels determine the degree of body adiposity. Nat Commun. 2023;14:7319. 37951979 10.1038/s41467-023-43080-x PMC10640548

[107] Masuda H , Fukumoto M , Hirayoshi K , Nagata K . Coexpression of the collagen‐binding stress protein HSP47 gene and the alpha 1(I) and alpha 1(III) collagen genes in carbon tetrachloride‐induced rat liver fibrosis. J Clin Invest. 1994;94:2481‐2488. 7989606 10.1172/JCI117617 PMC330081

[108] Kakugawa T , Yokota S‐I , Ishimatsu Y , et al. Serum heat shock protein 47 levels are elevated in acute exacerbation of idiopathic pulmonary fibrosis. Cell Stress Chaperones. 2013;18:581‐590. 23435730 10.1007/s12192-013-0411-5 PMC3745258

[109] Zhu J , Xiong G , Fu H , Evers BM , Zhou BP , Xu R . Chaperone Hsp47 drives malignant growth and invasion by modulating an ECM gene network. Cancer Res. 2015;75:1580‐1591. 25744716 10.1158/0008-5472.CAN-14-1027 PMC4401637

[110] Abd El‐Fattah EE , Zakaria AY . Targeting HSP47 and HSP70: promising therapeutic approaches in liver fibrosis management. J Transl Med. 2022;20:544. 36435779 10.1186/s12967-022-03759-z PMC9701392

[111] Wu S , Liang C , Xie X , et al. Hsp47 inhibitor Col003 attenuates collagen‐induced platelet activation and cerebral ischemic‐reperfusion injury in rats. Front Pharmacol. 2021;12:792263. 35082674 10.3389/fphar.2021.792263 PMC8784769

[112] Miyamura T , Sakamoto N , Kakugawa T , et al. Small molecule inhibitor of HSP47 prevents pro‐fibrotic mechanisms of fibroblasts in vitro. Biochem Biophys Res Commun. 2020;530:561‐565. 32747092 10.1016/j.bbrc.2020.07.085 PMC8387976

[113] Arkin MR , Tang Y , Wells JA . Small‐molecule inhibitors of protein‐protein interactions: progressing toward the reality. Chem Biol. 2014;21:1102‐1114. 25237857 10.1016/j.chembiol.2014.09.001 PMC4179228

[114] Kakugawa T , Mukae H , Hayashi T , et al. Pirfenidone attenuates expression of HSP47 in murine bleomycin‐induced pulmonary fibrosis. Eur Respir J. 2004;24:57‐65. 15293605 10.1183/09031936.04.00120803

[115] Tang S , Zhou S , Yin B , et al. Heat stress‐induced renal damage in poultry and the protective effects of HSP60 and HSP47. Cell Stress Chaperones. 2018;23:1033‐1040. 29779133 10.1007/s12192-018-0912-3 PMC6111100

[116] Wu D , Zhang M , Xu J , et al. In vitro evaluation of aspirin‐induced HspB1 against heat stress damage in chicken myocardial cells. Cell Stress Chaperones. 2016;21:405‐413. 26910344 10.1007/s12192-016-0666-8 PMC4837179

[117] Charlton M , Angulo P , Chalasani N , et al. Low circulating levels of dehydroepiandrosterone in histologically advanced nonalcoholic fatty liver disease. Hepatology. 2008;47:484‐492. 18220286 10.1002/hep.22063 PMC2906146

[118] Stewart J , Siavash H , Hebert C , Norris K , Nikitakis NG , Sauk JJ . Phenotypic switching of VEGF and collagen XVIII during hypoxia in head and neck squamous carcinoma cells. Oral Oncol. 2003;39:862‐869. 13679210 10.1016/s1368-8375(03)00110-6

[119] Cuenda A , Rouse J , Doza YN , et al. SB 203580 is a specific inhibitor of a MAP kinase homologue which is stimulated by cellular stresses and interleukin‐1. FEBS Lett. 1995;364:229‐233. 7750577 10.1016/0014-5793(95)00357-f

[120] Elias A , Crayton SH , Warden‐Rothman R , Tsourkas A . Quantitative comparison of tumor delivery for multiple targeted nanoparticles simultaneously by multiplex ICP‐MS. Sci Rep. 2014;4:5840. 25068300 10.1038/srep05840 PMC4894420

[121] Clichici S , Olteanu D , Nagy A‐L , Oros A , Filip A , Mircea PA . Silymarin inhibits the progression of fibrosis in the early stages of liver injury in CCl₄‐treated rats. J Med Food. 2015;18:290‐298. 25133972 10.1089/jmf.2013.0179

[122] Ali SO , Darwish HAEl‐M , Ismail NAEl‐F . Modulatory effects of curcumin, silybin‐phytosome and alpha‐R‐lipoic acid against thioacetamide‐induced liver cirrhosis in rats. Chem Biol Interact. 2014;216:26‐33. 24704557 10.1016/j.cbi.2014.03.009

[123] Tugues S , Fernandez‐Varo G , Muñoz‐Luque J , et al. Antiangiogenic treatment with sunitinib ameliorates inflammatory infiltrate, fibrosis, and portal pressure in cirrhotic rats. Hepatology. 2007;46:1919‐1926. 17935226 10.1002/hep.21921

[124] Honma S , Shinohara M , Takahashi N , et al. Effect of cyclooxygenase (COX)‐2 inhibition on mouse renal interstitial fibrosis. Eur J Pharmacol. 2014;740:578‐583. 24975097 10.1016/j.ejphar.2014.06.027

[125] Hsu Y‐C , Chiu Y‐T , Lee C‐Y , Wu C‐F , Huang Y‐T . Anti‐fibrotic effects of tetrandrine on bile‐duct ligated rats. Can J Physiol Pharmacol. 2006;84:967‐976. 17218962 10.1139/y06-050

[126] Zhao Y , Li H . Association of serum vitamin C with liver fibrosis in adults with nonalcoholic fatty liver disease. Scand J Gastroenterol. 2022;57:872‐877. 35189786 10.1080/00365521.2022.2041085

[127] Knüppel L , Ishikawa Y , Aichler M , et al. A novel antifibrotic mechanism of nintedanib and pirfenidone. inhibition of collagen fibril assembly. Am J Respir Cell Mol Biol. 2017;57:77‐90. 28257580 10.1165/rcmb.2016-0217OC

[128] Zhou B‐Y , Wang W‐B , Wu X‐L , et al. Nintedanib inhibits keloid fibroblast functions by blocking the phosphorylation of multiple kinases and enhancing receptor internalization. Acta Pharmacol Sin. 2020;41:1234‐1245. 32327724 10.1038/s41401-020-0381-y PMC7608201

[129] Yamakawa T , Ohigashi H , Hashimoto D , et al. Vitamin A‐coupled liposomes containing siRNA against HSP47 ameliorate skin fibrosis in chronic graft‐versus‐host disease. Blood. 2018;131:1476‐1485. 29363541 10.1182/blood-2017-04-779934

[130] Liu Y , Liu J , Quimbo A , et al. Anti‐HSP47 siRNA lipid nanoparticle ND‐L02‐s0201 reverses interstitial pulmonary fibrosis in preclinical rat models. ERJ Open Res. 2021;7:00733‐2020. 34109242 10.1183/23120541.00733-2020 PMC8181707

[131] Han X , Gong N , Xue L , et al. Ligand‐tethered lipid nanoparticles for targeted RNA delivery to treat liver fibrosis. Nat Commun. 2023;14:75. 36650129 10.1038/s41467-022-35637-z PMC9845313

[132] Lawitz EJ , Shevell DE , Tirucherai GS , et al. BMS‐986263 in patients with advanced hepatic fibrosis: 36‐week results from a randomized, placebo‐controlled phase 2 trial. Hepatology. 2022;75:912‐923. 34605045 10.1002/hep.32181 PMC9299674

[133] Kavita U , Miller W , Ji QC , Pillutla RC . A fit‐for‐purpose method for the detection of human antibodies to surface‐exposed components of BMS‐986263, a lipid nanoparticle‐based drug product containing a siRNA drug substance. AAPS J. 2019;21:92. 31332587 10.1208/s12248-019-0360-8

[134] Li ZN , Luo Y . HSP90 inhibitors and cancer: prospects for use in targeted therapies. Oncol Rep. 2023;49:6. 36367182 10.3892/or.2022.8443 PMC9685368

[135] Aalberse JA , Prakken BJ , Kapitein B . HSP: bystander antigen in atopic diseases? Front Immunol. 2012;3:139. 22666223 10.3389/fimmu.2012.00139 PMC3364480

